# Ecological and Physiological Studies of *Gymnodinium catenatum* in the Mexican Pacific: A Review

**DOI:** 10.3390/md8061935

**Published:** 2010-06-23

**Authors:** Christine J. Band-Schmidt, José J. Bustillos-Guzmán, David J. López-Cortés, Ismael Gárate-Lizárraga, Erick J. Núñez-Vázquez, Francisco E. Hernández-Sandoval

**Affiliations:** 1 Departamento de Plancton y Ecología Marina, Centro Interdisciplinario de Ciencias Marinas-Instituto Politécnico Nacional, A.P. 592, La Paz, B.C.S. 23000, Mexico; E-Mail: igarate@ipn.mx(I.G. -L.); 2 Centro de Investigaciones Biológicas del Noroeste, A. P. 128, La Paz, B.C.S. 23000, Mexico; E-Mails: jose04@cibnor.mx(J. J.B.-G.);dlopez04@cibnor.mx(D. J.L.-C.); enunez04@cibnor.mx(E. J.N.-V.);fhernan04@cibnor.mx (F. E.H.-S.)

**Keywords:** ecology, Gymnodinium catenatum, growth rate, harmful algae blooms, Mexican Pacific, paralytic toxins, physiological effects

## Abstract

This review presents a detailed analysis of the state of knowledge of studies done in Mexico related to the dinoflagellate *Gymnodinium catenatum*, a paralytic toxin producer. This species was first reported in the Gulf of California in 1939; since then most studies in Mexico have focused on local blooms and seasonal variations. *G. catenatum* is most abundant during March and April, usually associated with water temperatures between 18 and 25 ºC and an increase in nutrients. *In vitro* studies of *G. catenatum* strains from different bays along the Pacific coast of Mexico show that this species can grow in wide ranges of salinities, temperatures, and N:P ratios. Latitudinal differences are observed in the toxicity and toxin profile, but the presence of dcSTX, dcGTX2-3, C1, and C2 are usual components. A common characteristic of the toxin profile found in shellfish, when *G. catenatum* is present in the coastal environment, is the detection of dcGTX2-3, dcSTX, C1, and C2. Few bioassay studies have reported effects in mollusks and lethal effects in mice, and shrimp; however no adverse effects have been observed in the copepod *Acartia clausi*. Interestingly, genetic sequencing of D1-D2 LSU rDNA revealed that it differs only in one base pair, compared with strains from other regions.

## 1. Introduction

This review covers the state of knowledge of scientific studies of *Gymnodinium catenatum* Graham from the Gulf of California and the Pacific coast of Mexico. Many of the relevant studies have only been published in journals with low international impact, however this species is one of the most studied harmful algal blooms (HAB) species in Mexico. *G. catenatum* is a cosmopolitan species, occurring in the North Pacific, South East Pacific, Atlantic, Mediterranean Sea, South Caribbean Sea, East Arabian Sea, China Sea, South East Indian Ocean, and the Tasmanian Sea. This species is an unarmored dinoflagellate that occurs as chains and cysts, produces saxitoxin analogs, and forms HAB. It is distributed in warm-temperate regions and is a potential threat to other species including human health.

## 2. Distribution of *Gymnodinium catenatum* along the Western Coasts of Mexico and Associated Toxic Events

**Figure 1:**
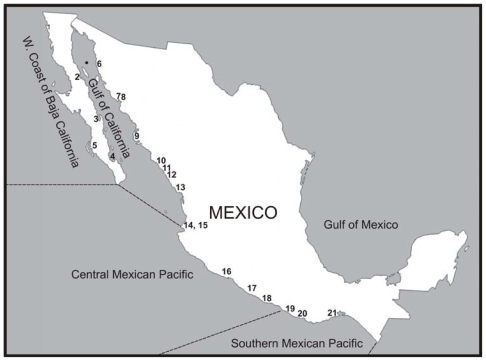
A map of Mexico showing coastal areas where *Gymnodinium catenatum* has been registered. Dark circle: first report; (1) Punta Colnett; (2) Bahía de Los Ángeles; (3) Bahía Concepción; (4) Bahía de La Paz; (5) Bahía Magdalena-Almejas; (6) Puerto Libertad; (7) Bahía Kun Kaak; (8) Bahía Bacochibampo; (9) Laguna de Macapule; (10) Cruz de Elota; (11) Punta Piaxtla; (12) Bahía de Mazatlán; (13) Teacapan; (14–15) Bahía Banderas and Puerto Vallarta; (16) Bahía de Manzanillo; (17) Lázaro Cárdenas; (18) Bahía de Acapulco;( 19) Laguna Corralero-Alotengo;( 20) Puerto Escondido;( 21) Salina Cruz.

In Mexico, *G. catenatum* has only been reported along the Pacific coast (Figure 1). Blooms of this species were first observed in 1939 in the northern central part of the Gulf of California [1,2]. *G. catenatum* is the only unarmored dinoflagellate in this genus that produces paralytic shellfish toxins (PST) [3].The production of PST was first demonstrated by Oshima *et al*. [4]. Probably the first record of paralytic shellfish poisoning (PSP) on the Mexican Pacific was in 1939 [5]. The first PSP linked to *G. catenatum* occurred from the coast of Sonora to Jalisco states in 1979, with a toxicity reaching up to 7,500 μg STXeq 100 g^−1^ [6]. Three deaths and 19 shellfish poisonings of humans occurred during this event; the victims that were most seriously affected were between five and 14 years old [6,7]. Since 1979, 10 cases of intoxication have occurred in this bay [8]. During the last few years, reports of HAB and presence of this species in several bays along the Pacific coast have increased ( Table 1).

In Mexico, studies of *G. catenatum* have focused mainly on local blooms. This species has a very marked seasonal pattern [15,16,54,55]. It is sometimes found from January through December, but observed most frequently during the months of March and April [3,30,54,55]. Blooms usually disperse in a few days or weeks [54], causing the sea to appear red, or alternatively may go unnoticed because higher cell aggregations often occur at depths of 10 to 20 m [56].

## 3. Ecological Studies

Nutrients play an important role in the relation of phytoplankton growth and distribution in aquatic ecosystems [57–59]. Eutrophication seems to be one of the principal reasons for the increase in frequency and the number of species causing HAB events as well as an increase in the duration of blooms [60,62–66]. Along the Pacific coast of Mexico, few studies have been done relating variations of nutrients with the presence or increase of HAB species. Most studies result from opportunistic observations; therefore, they lack physical and chemical data. In this section, we review the published information and, for the sake of clarity, the ecological studies are separated into four geographical regions: the Gulf of California, the west coast of the Baja California Peninsula, the central Mexican Pacific, and the southern Mexican Pacific (see Figure 1).

### 3.1. Gulf of California

The Gulf of California is a subtropical, semi-enclosed sea with exceptionally high primary productivity [67]. It supports important commercial fisheries, tourism, shrimp aquaculture, and has a high influence of nutrient inputs mainly coming from agriculture activities of the East coast [68]. Several bays are found in this area: Bahía de Los Ángeles, Bahía Concepción, Bahía de La Paz, Bahía Bacochibampo, and Bahía de Mazatlán. The biggest urban developments in this region are the cities of La Paz and Mazatlán. The hydrography and seasonal productivity in the Gulf of California is governed by winds, upwelling, and large-scale climatic events [65,69–73]. During the last few decades, the number of species and duration of HAB events in the Gulf of California has increased [12,54], with *G. catenatum* being one of the toxic species that frequently forms blooms.

Graham [1] found *G. catenatum* for the first time in samples collected in March 1939 in the northern part of the Gulf of California (~29° N), forming a visible bloom of ~1 × 10^6^ cells L^−1^ (Table 1). During the bloom, temperature and salinity ranged between 14–17 °C and 35.07–35.50 psu. More recently in this area, this species was reported in Puerto Libertad [9] and Bahía de Los Ángeles [11] (Table 1). In Bahía de Los Ángeles, paralytic shellfish toxins (PST) were detected in the scallop *Nodipecten subnodosus*(3–54 μg STXeq 100 g^−1^).

In Bahía Concepción (Figure 1), *G. catenatum* is often present without forming blooms and is abundant when the water column is stratified with high concentrations of nutrients localized primarily at the sub-surface level (~20 m) [56,74]. In this bay, Gárate-Lizárraga *et al*. [16] reported a temperature range between 22 °C and 26 °C for *G. catenatum* (Table 1) and suggested that the temperature is an important factor in outbreaks of this species. They also concluded that the mesotrophic process, characterized by nitrogen limitation, partly explains the high concentration of neoSTX in *G. catenatum*. The highest PST concentration reported in bivalve mollusks in this bay is of 298 μg SXT eq 100 g^−1^ in May 1999 [13]. Bahía Concepción is one of the few bays where studies on cyst dynamics have been done. Yields of cysts of *G. catenatum* are low, but seem to be a constant inoculum that sustains its population for long periods [75]. Cysts of this species have a short maturation period and can germinate under a wide range of environmental conditions [76,77].

In Bahía Bacochibampo(Figure 1), red tide events were monitored from 1970 through to 1994, with *G. catenatum* being one of the responsible species; however, no toxic events were reported [55] (Table 1). This bay is characterized by a high primary productivity associated with seasonal upwelling. In Bahía Kun Kaak (Figure 1), *G. catenatum* was reported during a multispecies bloom (April–May, 2003) that included a raphidophyte and other dinoflagellate species [10] and occurred under the influence of intensified upwelling and northwest winds. The raphidophyte dominated the bloom, which occurred at a mean temperature of 25.32 ± 0.99 °C, a salinity of 40.30 ± 1.03 psu, with phosphates and nitrates ranging from 0.54 to 3.0 mg L ^−1^ and 0.1 to 0.2 mg L ^−1^, respectively. In Bahía de La Paz (Figure 1), *G. catenatum*, has been registered several times since 1997 (Table 1), with cells densities varying from 1.6 × 10^2^ to 6.0 × 10^6^ cell L^−1^ within a temperature range from 18 to 26.5 ºC. In some events toxins have been detected in phytoplankton net samples and scallops, but toxin concentrations in scallops have never been above the maximum level for human consumption (0.14–67 μg SXT eq 100 g^−1^) [15]. In this bay, *G. catenatum* has also coincided with other bloom forming species. In June 2003, low cell densities (800–1,200 cells L^−1^) of this species were recorded during a bloom of *Chaetoceros debilis* Ehrenberg [18]. In February–March 2007, *G. catenatum* co-ocurred with *N. scintillans* [21], and during the bloom PST were found in several species of bivalve mollusks (Table 1). In June 2008, coinciding with a local upwelling event, Gárate-Lizárraga *et al*. [22] reported *G. catenatum* as one of the dominant species during a multispecies bloom of *M. rubra*, *Katodinium glaucum*(Lebour) Loeblich III, and *Gyrodinium instriatum* Freudenthal *et* Lee.

In June 2003, the presence of *G. catenatum* was associated with upwelling waters with high concentrations of nitrate, ammonium, and phosphate (4.5 μM, 7.4 μM, and 1.4 μM, respectively). However, these nutrient concentrations did not generate high *G. catenatum* densities, probably due to the dominance of the diatom. In June–July of 2006, another HAB event was recorded in Bahía de La Paz [19,20], under relatively low nutrient conditions. The average concentrations of nitrates, ammonium, and phosphates were low (1.0, 0.9, and 0.8 μM, respectively) [20].

Along the coastal lagoons of Sinaloa (eastern shore of the Gulf of California), *G. catenatum* is a common bloom forming species [78,79]. The first HAB reported in Mexico of this species was in Bahía de Mazatlán (Figure 1) from February to April of 1979. The bloom was very intense and extensive, with average cell densities of 1.2 × 10^6^ cells L^−1^ and high levels of PST in bivalves (Table 1). Since this event, this area has been one of the most extensively monitored in our country. Between 1981 and 2006, many blooms of *G. catenatum* have been reported, however toxin analyses have not always been reported. From 2003 to 2007, PST in mollusks were between 63 and 1315 μg SXTeq 100 g^−1^[30]. Analysis of the HAB of *G. catenatum* occurring in this area demonstrate that these events occurred mainly in late winter and early spring [79], during upwelling events [6,12,25,28,32,78,80–84], and within a temperature range of 16.5 to 32.9 ºC [9,15,23]. The effect of the El Niño or La Niña event for this species is not clear since Alonso-Rodríguez and Ochoa [80] did not find any bloom of *G. catenatum* during La Niña 2000 in Bahía de Mazatlán, however in Bahía de Bacochibampo, blooms of *G. catenatum* were found to increase during a La Niña events[ 55].

In Bahía de Mazatlán, *G. catenatum* has also been reported with other bloom forming species [28,82]. Alonso-Rodríguez [28] related a multispecies bloom (February 1995 to August 1996) with wind mixing processes, which contributed to the resuspension of cysts and nutrient increases, however, there was no clear relationship to eutrophication. In February 1996, an abrupt decrease of 2.5 °C in the mean surface temperature (from 24.5 °C to 22 °C) followed by a rapid rise in temperature to 24.0 °C, over a three day time period, coincided with a bloom of *G. catenatum* [83], suggesting that the temperature change favored the growth of this species. Recently, in Laguna de Macapule, a coastal lagoon in Sinaloa, *G. catenatum* was reported with an abundance of 38.8 × 10^3^ cell L^−1^ [32]. Clearly, Bahía de Mazatlán is one of the zones in Mexico with the highest number of studies on the presence of red tide events caused by *G. catenatum*. Palynological records show that *G. catenatum* cysts have been present in the Gulf of California since ~1483 [81], with higher abundances from 1888 to 1920. Cyst abundances seem to increase during La Niña conditions and decrease during warmer El Niño events, abundances were also inversely related with sea surface temperature (SST), decreasing steadily from 1972 to 1994 as the SST increased in this area.

### 3.2. West Coast of Baja California Peninsula

The western coast of the Baja California Peninsula is influenced by the California and North-equatorial Currents [83], and El Niño events [86,90]. It has a low human influence that is mainly linked to local fisheries of sardines and mollusks [88]. Bahía Magdalena, found in this area (Figure 1), is a highly eutrophic ecosystem influenced by intense currents, mixing processes, and upwelling [89]. Red tides have been reported during coastal upwelling [90]. In this bay, *G. catenatum* has been recorded once in net phytoplankton samples [34] (Table 1). This species has also been found in low concentrations (1200 to 4200 cells L^−1^) near Punta Colnett, during summer when regional upwelling is dominant [33].

### 3.3. Central Mexican Pacific

This area is an important tourist zone, and recently aquaculture activities—mainly shrimp farms—have been developed. The principal bays in this area are Bahía Banderas and Bahía de Manzanillo. In Bahía de Manzanillo the main merchant ship commerce is found [91]. Eutrophication linked to tuna factories [38] and continental nutrient discharges have been observed [92].

In Puerto Vallarta, *G. catenatum* was reported for the first time in 1979 (Table 1), during the same event that extended from La Cruz de Elota, Sinaloa to Jalisco [6]. In the winter and spring of 1999, a bloom of *G. catenatum* lasted approximately three months covering a large part of Bahía de Manzanillo [40,41] (Table 1); high oxygen values (18 mg L^−1^) and low nitrate concentrations were recorded (0.05 μg-at L^−1^). Seasonal marine currents in the area seemed to have dispersed the biomass, which avoided its accumulation at the bottom, which would create anoxic conditions that may limit the length of the bloom [93].

During 2000 and 2001, HAB of *G. catenatum* were documented in Puerto Vallarta and Bahía de Banderas (Figure 1) [35–37]. During spring 2007, *G. catenatum* abundances between 450 to 2,134,000 cells L^−1^; however toxin levels found in the oyster species *Crassostrea iridescens* Hanley were below the maximum limit for human consumption (Table 1) [37]. In 2007, toxin concentration in mollusks was high (235 μg STXeq 100 g^−1^) [42]. In a recently monitored area (Lázaro Cárdenas, Michoacán), a *G. catenatum* bloom was reported for the first time in November 2005, with a cell density of 560,000 cells L^−1^[ 94].

Several hypotheses have been proposed for this area to explain the presence of these blooms: eutrophication linked to tuna factories [38], continental nutrient discharges [89], and transportation of cysts by ballast water [38]. Further detailed studies need to be carried out to confirm these hypothesis.

### 3.4. Southern Mexican Pacific

In this area important tourist influence is observed with the concomitant impact in nutrient inputs. Also river drainage is an important source of nutrients [95,96]. The principal bays are Bahía de Acapulco, Laguna Corralero-Alotengo, and Bahías de Huatulco.

Bahía de Acapulco (Figure 1) is a shallow bay (average depth, 20 m) with high anthropogenic impact and land drainage [95,96]. In March–April 1999, a red tide of *G. catenatum* was registered for the first time in Bahía de Acapulco (Figure 1), with cell densities between 7.6 × 10^3^–37.6 × 10^3^ cells L^−1^, despite these relatively low cell densities, toxin concentrations in mollusks were above the maximum limit for human consumption [45,46] (Table 1). This species appeared again in November 2005 (6.29 × 10^3^ cells L^−1^), January 2006 (10 × 10^6^ cells L^−1^), and December 2007 (1942 × 10^3^ cells L^−1^) [47,97]. These *G. catenatum* abundances are the highest reported for the Mexican Pacific. Toxicity values in mollusks during these events varied between 25 and 1152 μg STXeq 100 g^−1^(Table 1) [48].

Further south, in the coasts of Oaxaca, blooms of *G. catenatum* have occurred since 1989 [50] (Table 1). Cell abundances have varied from 13 × 10^3^ to 10 × 10^6^ cells L^−1^ [38,51,52]. Paralytic toxins were above the maximum limit for human consumption in 2001, however toxicity was also related to the presence of *Pyrodinium bahamense* [53,98], another PSP toxin producer.

## 4. Grazing Studies

There are few records on the effect of grazing activity on *G. catenatum* under natural conditions. Alonso-Rodríguez *et al*. [99] observed *G. catenatum* cells in *Noctiluca scintillans* vacuoles during a HAB of *G. catenatum*. Predation activity of *G. catenatum* by *N. scintillans* may be facilitated by the high swimming velocity of *N. scintillans*.

Grazing of *N. scintillans* on cells of *G. catenatum* (chains of 4–16 cells per *Noctiluca*) has also been observed in Bahía de Los Angeles, Bahía Concepción, and Bahía de La Paz [47]. *In vitro* studies have confirmed an important grazing activity of *N. scintillans* towards *G. catenatum* [100]. The copepod *Acartia clausi* Giesbrecht also had a high grazing rate on *G. catenatum*, with no visible short time harmful effects on the copepod [101]. These data suggest that *N. scintillans* grazing can be an important factor in controlling *G. catenatum* blooms.

## 5. Toxicity Studies

### 5.1. PST in Phytoplankton Samples Related to the Presence of G. catenatum

Toxin analyses from phytoplankton net samples is a method to confirm that a toxic organism is found in the plankton community, and can help us understand the toxin profile of a toxic species in the environment. The toxin profile in net phytoplankton samples for Bahía Concepción, Bahía de La Paz, and Bahía de Mazatlán has been variable, however STX, neoSXT, GTX2-3, dcGTX2-3, B1, C1, and C2 have been found [15,16,21] (Table 2). Decarbamoyl (dcGTX2 and dcGTX3) and *N*-sulfocarbamoyl (C1 and C2) are usually the toxins with a high molar contribution [15,21]. Average toxin content reported in field phytoplankton samples from Bahía Concepción varied from 3.8 to 639.1 ng PSP filter^−1^, and in Bahía de La Paz from 4.32 to 90.54 ng PSP filter^−1^, Differences in the toxin profile have been observed between sampling stations in net phytoplankton samples, despite being collected during the same event; these differences could be explained by the different development stages of the red tide [21]. More data are needed from field samples in order to explain these differences.

### 5.2. Toxin Content and Toxin Profile of G. catenatum S trains

Very limited data exists on the toxin content of natural populations of *G. catenatum*. Gárate-Lizárraga *et al*. [102] and Band-Schmidt *et al*. [103,104], found that the toxin content of the *G. catenatum* strains of the Gulf of California was higher (average values of 25.7–101 pg STXeq cell^−1^) than the toxin content of natural populations (1.01 pg STXeq cell^−1^). This could be related to strain growth conditions, because, in culture, the nutrient concentrations are much higher than in the environment. However, more data on toxin content per cell under natural conditions needs to be obtained to confirm these differences.

Differences found in the toxin profile of *G. catenatum* strains *in vitro* can be explained partly by the culture medium used. For instance, when using modified f/2 media, Bahía Concepción strains produce dcSTX, dcGTX2-3, C1, and C2 (Table 3). Other toxins, such as neoSTX, GTX2-3, B1, and B2 are only present in some strains and in low molar percentage (below 3 mol%) [17]. In contrast, when using GSe media, the number of saxitoxin analogs is higher (STX, neoSTX, dcSTX, dcGTX2-3, B1-2, C1, C2, C3, and C4); and the contribution of neoSTX is higher (from 6–46%) [104]. Neosaxitoxin has not been reported for strains from *G. catenatum* of other regions.

Differences in the toxin profile have also been observed with strain origin [102,104]: Bahía Concepción strains had the highest content of C1; BAPAZ and BAMAZ strains had a higher percentage of neoSTX. Differences in the toxin composition with culture age were observed only in BAMAZ and BAPAZ strains. These differences with culture age seem to be related to chain length, since cultures with a higher percentage of long chains had more neoSTX, while those with a higher proportion of short chains had a lower concentration of neoSTX. Differences in toxicity per cell were also observed:BAPAZ and BAMAZ strains were the most toxic (101 pg STXeq cell^−1^), whereas strains from BACO were the least toxic (13 pg STXeq cell^−1^).

The most abundant toxins in phytoplankton samples (dcSTX, dcGTX2-3, C1, and C2) do not vary in concentration in response to changes in culture media, strain origin, and N:P ratios. For example, when cultivating a strain from BACO with N:P ratios ranging from 1:6 to 32:1, no observable differences were found in the toxin profile across the different treatments [105]. However, the production of neoSTX often varies with the strain origin and with different culture media (Table 3). Additionally, the culture age seems to play a role in a differential production of saxitoxin analogs of *G. catenatum* [104,105]: after the tenth day of growth with different N:P ratios, an increase in the percentage of carbamoyl and decarbamoyl toxins occurred (18–26% and 11–16%, respectively) as compared with the toxin composition during the first eight days of culture, with a carbamoyl production from 9 to 14% and decarbamoyl production below 5% [105].

It seems that strains from the Gulf of California are characterized by the presence of neoSTX, and they seem to have evolved particular physiological responses to their environment that are reflected in their toxin profiles, suggesting different populations. Also, the variation in the toxin profiles of *G. catenatum* isolated from different zones of the Mexican Pacific (Bahía Concepción, Bahía de Mazatlán, and Bahía de La Paz), could be related to the differences in the source and concentration of nutrients of each embayment [102].

### 5.3. Presence of PST in Mollusks Linked to HAB of G. catenatum

The analyses of the toxin content in different clams and scallops of several embayments from the Gulf of California has been done during the presence of *G. catenatum* (Table 1). Toxicity was variable: the highest toxicity values in mollusks were found in Bahía de Mazatlán (up to 7,500 μg STXeq 100 g^−1^) [6] (Table 1).

The toxin profile of mollusks feeding naturally with wild populations of *G. catenatum* has also been determined in several species [15,21,30,37,103] (Table 4). The toxin profile varied within each zone and with mollusk species. In general, mollusks usually contained a high molar percentage of C1 and C2 toxins (see Table 4), similar to cultured *G. catenatum* strains, and phytoplankton net samples. Decarbamoyl toxins (dcSTX, dcGTX2-3) were also found in high molar percentages in mollusk samples.

An annual variation of toxicity and toxin profile in marine bivalves has been performed in Bahía de Los Ángeles, Bahía Concepción, Bahía de La Paz, and Bahía de Mazatlán [11,13,15,17,30,34]. Toxicity levels were correlated to the presence and abundance of *G. catenatum* cells in the water column, showing a clear seasonal pattern with higher toxin content in mollusks in May–June [13,17]. Toxin profiles of PST varied monthly, probably according to the cell abundance and metabolism of the mollusk. In most cases, *N*-sulfocarbamoyl toxins were the most abundant toxins, contributing usually more than 60% of the total toxin content. These high molar percentage contributions of the *N*-sulfocarbamoyl toxins may explain the low toxicity found in the shellfish (Table 2). Most shellfish contain a mixture of several PST, depending on the species of algae, geographic area, and shellfish species involved. For instance, toxic shellfish that grow in cold or temperate waters usually contain sulfated C toxins, GTX2-3, and STX [106]. When bivalves have recently ingested toxin-containing dinoflagellates, they typically contain high proportions of C1–C2 [4,13,15,107]. Experimental studies of toxicity in the scallop species *M. squalida* fed with *G. catenatum* also presented a high percentage of *N*-sulfocarbamoyl toxins, supporting the hypothesis that toxicity in scallops from the Gulf of California are linked to this dinoflagellate [108].

The presence of PST (GTX-2 and C1) has also been found in the liver of puffer fish *Sphoeroides annulatus* Jenyns from Bahía de La Paz and the mucus of *Arothron meleagris* Laceepéde from Punta Pericos. The analyses of the feeding behavior of these organisms, and the existence of PST dinoflagellates (e.g. *G. catenatum*) in the zone suggest the transfer of these toxins via mollusks[109].

## 6. Toxic Effects of *G. catenatum* on Terrestrial and Marine Organisms or Toxic Effects of *G. catenatum* on Other Organisms

### 6.1. Laboratory Studies

Experimental work utilizing *G. catenatum* has focused on diverse physiological aspects of mammals, crustaceans, and bivalves (Table 5). Necropsy in mice (Swiss CD1 and BALB/c) with an acute saxitoxin exposure show a pronounced ischemic zone in the liver border. A degeneration of Purkinje cells in the cerebellum was also observed in histological observations of mice exposed to toxic extracts of *G. catenatum* [110]. Effects on grazing rates, egg production, and hatching success when the copepod *Acartia clausi* was fed with the dinoflagellate [101], showed no apparent harmful effects. However, egg production and hatching success increased with a higher consumption of *G. catenatum*. In the same experiment, the toxin profile of the copepod was analyzed, finding neoSXT, dcSTX, dcGTX2-3, B1-2, and C2 with a toxicity value of 12.7 pg STXeq copepod^−1^ [16]. In conclusion, *A. clausi* not only accumulates PST but can also transform them. In natural populations, the effect on white shrimp larvae *(Litopenaeus vannamei* Boone) was lethal at concentrations of 50,000 cells L^−1^ [99] (Table 1). Juvenile and adult shrimps injected with varying quantities of PST showed a time-to-death below seven minutes [111]. Chronic assays in shrimp also demonstrated significant differences in survival rates, percentage of feed, and weight gain [112]. Gastric glands and muscle of shrimp retained PST for a longer period and histological damages were observed in the heart, gastric gland, and brain tissue. These effects may explain the relationship of shrimp nauplii and postlarvae mortality in farms during a bloom of *G. catenatum* [23]. 10–20% mortality was observed in adults and metanauplii in *Artemia* exposed to *G. catenatum*. Behavioral symptoms such as erratic swimming, spasm, and convulsions were observed in *Artemia* when exposed to *G. catenatum* cells. Mortality of *Artemia* exposed to *G. catenatum* has been observed previously [113], and it has been demonstrated that *Artemia* can transfer PST via the marine food chain (*Alexandrium tamarense* to *Artemia salina* to *Neomysis awatschensis* to *Lateolabrax japonicus*)[114].

STX and neoSTX accumulated in the bivalves, *M. squalida* and *Nodipecten subnodosus* Sowerby, when exposed to *G. catenatum* cultures [108,115]. Differences in the toxin profile of the *G. catenatum* strains used in these studies were observed; the strain used by Estrada *et al.* [115] was rich in gonyautoxins, while the toxin profile of the strain used by Pérez-Cruz [108] was composed of STX, neoSTX, dcSTX, dcGTX2-3, C1, and C2, which is similar to those reported previously [17]. These differences could be due to culture conditions of the dinoflagellate, culture age, strain differences or extraction methods used. In both mollusks, the presence of highly toxic analogs suggests a biotransformation process [117] in a short time from *N*-sulfocarbamoyl to carbamoyl toxins. The depuration rate was moderate, between 0.19 and 0.23 day in the clam [108] and 0.4 day in the scallop [115], the toxin content after the thirteenth day in clams was only 4–5% of that found at the time of initiation. Short-term effects (24 h) on the immunological system of the clam *N. subnodosus*, when fed with *G. catenatum*, were also studied by Estrada *et al*. [118,119]. Several enzymes involved in antioxidant, lipid peroxidation, and hydrolytic activity were considered. Important changes were noted in the exposed scallops with an increase of antioxidant and hydrolytic enzymes mainly in the gills and the digestive gland. A melanization in gills, mantle, and labial palps was also reported. Lipid peroxidation has also been observed in *Dosinia ponderosa* and *Crassostrea gigas* exposed to *G. catenatum* cells [120]. Different responses to toxin exposure can be expected, since life history exposure to toxins plays an important role [116].

### 6.2. Aquaculture Activities

Blooms of *G. catenatum*, among other toxic species along the coast of Sinaloa (Gulf of California), caused the death of nauplii and adult shrimps in shrimp farms [23,24,121]. These authors assumed that the toxicity was caused by *G. catenatum* in the water introduced to the ponds by the pumping system. The mortality occurred with the events of HAB of *G. catenatum* from February to March 2001, concluding that the contamination, climatic conditions, and inadequate management in fertilization, feeding rate, and food composition can provoke delay in the growth of the shrimp and decrease their production through massive mortality. Sierra-Beltrán *et al*. [122] speculated that in the central part of the Gulf of California, the urban aquatic residues and the eutrophication generated by the shrimp farmers could be responsible for the proliferation of *G. catenatum* and other species. García-Hernández *et al*. [10] concluded that the residues of the shrimp farms and shrimp larvae producing laboratories are deposited without treatment to Bahía Kun Kaak, which is known to be a highly productive ecosystem. This natural condition, and the water input with high content of nitrogen and phosphorus compounds, probably contributes to the formation of red tides. Other regions of the coasts of Sonora (Gulf of California) have also had high nitrogen contributions, related to fertilization process, which are added to coastal waters by runoff (36.8 × 10^6^–201 × 10^6^ moles of N) [64]. These findings suggest that this loss by irrigation can support phytoplankton blooms in the Gulf of California. It is probable that the eutrophication processes in this ecosystem are seasonal events [123] and are influenced by upwelling events, agriculture, and aquaculture contributions. Dumping and wastewater treatment regulations are recommended to obtain a good water quality, and equilibrium in the phytoplanktonic communities. These results show the importance of establishing continuous monitoring of the water quality that flows into and out of shrimp culture systems.

## 7. Growth Variations of *G. catenatum*Strains of the Gulf of California

Growth rates of different *G. catenatum* strains vary from 0.08 to 0.82 day^−1^ (Table 6), with the highest exponential growth rates obtained in GSe media with values above 0.70 day^−1^. Maximum cell densities vary between 1,090 and 3,940 cells mL^−1^ and are usually obtained between 14 and 18 days of growth.

Growth rate varies significantly with temperature. In a strain from Bahía Concepción, the highest growth rates (0.18–0.21 day^−1^) are obtained between 21 and 30 ºC. A high salinity range (15–40 ups) is also observed in Bahía Concepción strains, with the highest growth rates (0.30 day^−1^) occurring at salinities from 28–38 ups with seawater from Bahía Concepción. The optimal temperature and salinity ranges coincide with the temperatures and salinities at which *G. catenatum* has been reported in different regions of the Mexican Pacific, with the exception of the Northern Gulf of California where *G. catenatum*has been reported at lower temperatures (see ecological studies).

## 8. LSUrDNA Sequences

PCR amplifications of Bahía Concepción strains of the D1-D2 fragment of the nuclear large subunit rDNA gene resulted in a single product of approximately 889 base pairs [124]. Strains from Bahía Concepción present a constant characteristic at position≈453, a single nucleotide polymorphism was observed, presenting cytosine instead of guanine. This single base polymorphism could indicate a mutation or genetic isolation from other *G. catenatum* populations. This possible genetic isolation or population differentiation could be explained by the hydrographic conditions mentioned previously for Bahía Concepción.

## 9. Conclusions

In summary, *G. catenatum* produces PST, is distributed along the Mexican Pacific coast, and has been related to the presence of PST in mollusks. Scarce reports exist on the physical and chemical conditions in this coastal ecosystem associated with blooms of *G. catenatum*. Nevertheless, from the available information, we can conclude that this species tolerates wide temperature and salinity ranges, and N:P ratios which probably has allowed its distribution along the Mexican Pacific. Its toxicity has been related to nutrient availability. Its capacity to produce PST and its environmental and human health costs has directed more attention towards the study of this species, increasing the number of published records in recent years as well as records in different regions of the country. However, many of these publications remain as thesis and/or have been published in Spanish journals, thus limiting their access for international colleagues. A high percentage of these blooms have been associated with an increase in the nutrient contribution, mainly by nitrogen compounds from upwelling events or transitional periods in the water column, and with low SST. In many occasions, *G. catenatum* has been found with other bloom forming species. Future investigations need to focus on the evaluation of the eutrophication process with systematic monitoring that can allow the quantification of the alterations in the organic matter balance, inorganic nutrients, and the interaction of different species associated with the presence of *G. catenatum*. In addition, more attention needs to be directed to understand the effect of grazers and their possible role on the development or regulation of HAB of this species.

The toxin profile found in net phytoplankton samples, shellfish, and *G. catenatum* strains of the Gulf of California is variable, however a common characteristic is that dcGTX2-3, dcSTX, C1, and C2 are always present. Unfortunately, many programs monitoring HAB events of *G. catenatum* in our country do not have the possibility to determine toxin content in mollusks, and so far, no studies have been done on the benzoate and deoxy decarbamoyl type toxins.

In some regions, data on the toxin analyses of phytoplankton net samples have proved to be useful in the monitoring activities of HAB of *G. catenatum*, as an early detection method for planktonic toxin producing organisms. More data on the toxin content per cell of field samples of *G. catenatum* needs to be obtained. However, latitudinal differences have been observed in the toxicity and toxin profile in cultured strains from different embayments of the Gulf of California. So far strains from Bahía de Mazatlán have a higher toxicity and a higher content of carbamoyl toxins than northern strains of the Gulf of California. Interestingly, field samples of *G. catenatum* show a less complex toxin profile than cultured strains. Further work needs to be done to understand the relationship between environmental factors and toxin variability.

Laboratory studies show that Mexican *G. catenatum* strains produce effects in most of the organisms tested. Diverse histological and immunological effects were evident in shrimp, mollusks, and mice. However, in a short term study no adverse effects were observed in the copepod *A. clausi*, when fed with this dinoflagellate. It has been stated that the copepod and *Noctiluca* could play a key role in controlling the occurrences of red tides of this species. Clearly, more research must be done to evaluate the role of *G. catenatum*in the ecosystem and aquaculture activities of the Mexican coasts.

The genetic sequence of the D1-D2 LSU rDNA differs from sequences of the same region in strains examined from Europe, Asia or Australia. This suggests that this species in the Gulf of California is not an introduced species, and could be used as a genetic marker for this population. At the moment, the design of DNA probes for the detection of *G. catenatum* in water samples is being carried out. This is supported with palinological studies that have demonstrated the presence of *G. catenatum* in this region since ~1483 [25].

Despite the toxicity of *G. catenatum* and its wide distribution within most regions of the Mexican Pacific, there has been low monitoring effort, and probably many events have gone unnoticed. The increase in the reports of this species during the last decades is probably due to the increased interest in HAB events of this species, and the number of colleagues researching HAB. In spite of being one of the most studied toxic dinoflagellate species in Mexico, there are still many research areas that have not been addressed, such as a finer monitoring design, definitions and quantification of physical-biological cell interactions, interactions between species, cyst studies (transportation, distribution), diverse toxic effects on a wider number of taxa, and toxin metabolism.

## Figures and Tables

**Table 1:** Reports of *Gymnodinium catenatum* along the Pacifi ccoast of Mexico.

Region	Year	Locality	Abundance (cells L^−1^)	Toxicity (μg STXeq per 100 g^−1^) and bivalve species	Temperature (ºC)	Ref
Gulf of California	1939	N Gulf of California	1 × 10^6^	nd	14.0–17.0	[1]
	1981	Puerto Libertad	190 × 10^3^	nd	nd	[9]
	2003	Bahía Kun Kaak	Nd	nd	25.32 ± 0.99	[10]
	2006	Bahía de Los Ángeles	Nd	3–54 *Nodipecten subnodosus*	16.0–23.0	[11]
	1990	Bahía Concepción	1.8 × 10^2^–3 × 10^3^	nd	nd	[12]
	1999		5.7 × 10^5^	298 *Argopecten ventricosus*	18.0–25.0	[13]
	2000		500–4 × 10^4^	63 *A. ventricosus*	18.0–25.0	[13,14]
	1997–1998	Bahía de La Paz	1.60 × 10–2.6 × 10^2^	0.14–5.46 *Megapitaria squalida*	18.0–26.0	[15]
	2001		Nd	2–67 *A. ventricosus* PST in net phytoplankton samples	22.0–26.0	[16,17]
	2003		1–1.20 × 10^3^	nd	18.0–22.0	[16,18]
	2006		1.0–3.6 × 10^3^	3–4.5 *M. squalida* 4–9 *Dosinia ponderosa*	18.5–26.5	[19,20]
	2007		6–2.39 × 10^6^	0.40–37.74 *M. squalida, M. aurantiaca, D. ponderosa. Modiolus capax, Pinna rugosa, P. multicostata*	20.0–21.9	[21]
	2008		8–79 × 10^3^	nd	20.0–24.0	[22]
	1995–1996	Shrimp culture pond, Sinaloa	?	?	?	[23]
	1997		15 × 10^3^	40 *Crassostrea iridescens*. Nauplii and adult mortality of *L. vannamei*	nd	[15,24]
	2001		18–528 × 10^3^	29 oyster Nauplii mortality of *L. stylirostris.* No toxins detected in nauplii	nd	[23]
	1979*	Cruz de Elota, Punta Piaxtla, Bahía de Mazatlán, and Teacapan, Sinaloa	240–6.6 × 10^6^	〈20–7640 *C. iridescens* and *Donax* sp. extensive fish	21.60	[7,25,26]
	1981	Bahía de Mazatlán	35–544 × 10^3^	nd	19.74–20.52	[9]
	1985		65 × 10^3^	nd	22.04	[9,27]
	1986		170–940 × 10^3^	nd	20.64–22.34	
	1988**		1000 × 10^3^		20.94	
	1994–1995		1.2–2.2 × 10^5^	nd	21.14–22.54	[28]
	1996		3856–5000 × 10^3^	nd	21.0–32.9	[23]
	1997		3856–5000 × 10^3^	〈35 *Ostrea iridescens*	nd	[29]
	2001		1.5–196 × 10^3^	39.40 *C. iridescens*	16.5–25.0	[9,15]
	2003–2004		71–115 × 10^3^	63–1315	19.0–24.0	[30]
	2006		148 × 10^3^	nd	21.2–22.7	[31]
	2005	Laguna de Macapule	38.8 × 10^3^	nd	nd	[32]
W. Coast of B. California Peninsula	1996	West Coast of Baja California	1.2–4.2 × 10^2^	nd	13.0–17.0	[33]
	2005–2006?	Bahía Magdalena-Almejas	Presence in net phytoplankton samples	negative *Anadara tuberculosa*	nd	[34]
Central Mexican Pacific	1979	San Blas and Puerto Vallarta	Nd	〈20 *Crassosstrea cortesiensis*	nd	[25,35]
	2005	Bahía de Matachén	1010 × 10^3^	nd	nd	[36]
	1999	Bahía de Manzanillo (Puerto Interior)	2.5–3.8 × 10^6^	11–13 *C. iridescens*	nd	[37,38]
	1989	Bahía de Manzanillo and Santiago	5000 × 10^3^	nd	nd	[39]
	2002		832 × 10^3^	nd	nd	
	2000	Bahía de Manzanillo	>3500 × 10^3^	235 Oyster	23.0–25.0	[40,41]
	2007	Bahía Banderas	3.53 × 10^3^–3.8 × 10^6^	29–235.28 *C. iridescens*	nd	[42,43]
	2001		450–2134 × 10^3^	11–13 *C. iridescens*	23.0	[37, 44]
	1999	Bahía de Acapulco	0.01–78 × 10^6^	120–209 *O. iridescens*	nd	[45,46]
	2005		6.29 × 10^3^	25–217	nd	[47]
	2006		10 × 10^6^	112	nd	[47–49]
	2007		3 × 10^3^–13 × 10^6^	1152	nd	
South Mexican Pacific	1989	Salina Cruz to Chiapas	Nd	Presence of PSP	nd	[50]
	1998	Puerto Escondido to Huatulco	4–10 × 10^6^	〈80	nd	[51,52]
	2001	Laguna Corralero-Alotengo, Oaxaca	Nd	24–1456 Mussel	nd	[53]
	2006	Gulf of Tehuantepec, Coasts of Salina Cruz	13 × 10^3^	nd	nd	[38]

nd, not determined;

* 19 people intoxicated with three human deaths;

** 10 intoxicated people.

**Table 2:** Paralytic toxin profile in phytoplankton samples from different embayments of the Gulf of California.

Toxin	Bahía de Mazatlán	Bahía Concepción									Bahía de La Paz			
	2001 Apr	2001 May	2002								2002			2007
			18 Jan	30 Jan	14 Feb	6 Mar	17 Apr	2 May	21 May	8 Jul	15 Mar	12 Mar	20 Aug	7 Mar
STX		64.3	-	-	-	-	1.0	-	3.2	-	34.6	-	1.4	0–31
neoSXT	-	-	-	-	-	-	-	-	7.8	-	-	-	-	0–25
GTX2	-	-	-	-	-	-	-	-	9.0	-	-	-	-	0–5.4
GTX3	-	-	-	-	-	-	-	-	2.6	-	-	-	-	0–3.6
dcSTX		17.4									9.0	62.9	8.3	-
dcGTX2	22.6	2.0	78.2	47.3	88.6	73.9	66.8	70.2	1.6	72.8	37.0	37.1	52.5	-
dcGTX3	27.4	3.6	21.8	25.8	11.4	26.1	18.5	29.8	0.4	27.2	12.0	-	18.5	0–1.1
B 1		4.4	-	-	-	-	0.8	-	-	-	7.4	-	5.7	0–5.4
B 2	-	1.0									-	-	-	-
C 1			-	16.0	-	-	9.4	-	62.6	-	-	-	10.5	0–37.0
C 2	50.0	7.4	-	10.9	-	-	2.5	-	12.8	-	-	-	3.2	53.8–68.9
	1.5 ng STXeq cell^−1^													

nd = no data. References [15–17,21].

**Table 3:** Average toxin profile (% mol) of *Gymnodinium catenatum* strains isolated from the Gulf of Californiaunder different growth conditions.

Strain Origin	Media	STX	neoSTX	GTX2	GTX3	dcSTX	dcGTX2	dcGTX3	B 1	B 2	C 3	C 1	C 2	C 4
BACO	GSe	0–0.6	6.6–12.1	nd	nd	2.1–2.8	3.3–4.7	0.9–1.4	0.7–0.9	3.7–7.6	1.6–2.9	50.9–55.9	18.6–22.8	0.1–0.6
BAPAZ		0–0.8	25.7–35.2			6.3–24.2	1.6–2.9	0.5–0.9	0.4–0.8	4.1–12.9	1.6–7.1	19.7–33.1	7.0–15.2	0–2.7
BAMAZ		0.1–0.6	29.2–46.3			1.1–3.4	2.2–3.8	0.7–1.4	0.2–0.4	7.5–15.3	2.3–4.3	23.7–31.7	8.6–13.5	0.5–1.7
BACO	f/2	nd	0–5.1	0–0.3	0–0.1	19.4–43.2	19.3–43.1	6.5–11.7	0–0.2	0–1.5	nd	12.8–39.9	4.6–38.7	nd

BACO, Bahía Concepción; BAPAZ, Bahía de La Paz; BAMAZ, Bahía de Mazatlán. nd, not detected. References [17,104].

**Table 4:** Toxin profile (mol%) in different mollusk species related to the presence of *Gymnodinium catenatum.*

Toxin	Bahía Concepción *Argopecten ventricosus* May 99, 00, 01	Bahía de Mazatlán *Crassostrea iridescens* April, 01	Bahía de La Paz *Megapitaria squalida* Dec, 01–Aug, 02
STX	0–0.92	-	0–38.69
neoSXT	0–9.00	5.71	-
GTX2	0–31.17	-	0–41.19
GTX3	0–4.79	0.89	0–16.02
dcSTX	0–41.62	18.54	0–62.90
dcGTX2	0–40.67	2.52	0–52.45
dcGTX3	0–59.33	3.21	0–39.77
B 1	0–42.34	9.80	0–7.40
B 2	0–1.94	1.91	-
C 1	0–54.61	37.89	0–52.54
C 2	0–47.72	19.50	0–35.07
C3	0–3.32	-	-
C4	0–3.98	-	-

References [15,17].

**Table 5:** Toxin profile of *Gymnodinium catenatum*strains and their effect on different organisms.

Organism tested		Strain	Culture conditions	Total toxicity (pgSTXeq cell^−1^ or μg eq. STX)	Toxin profile	Effects	Ref.
Mammals	Mouse model, *Mus musculus* (BALB/c and CD1mice)	GCCV-6	f/2+Se, 33 ‰_,_ 25 ± 1 ºC, 12:12 L:O, 150 μEm^2^ s^−1^, Fernbach flasks	0.2, 0.3 μg eq.STX	STX, dcSTX GTX-2,3 dcGTX-2,3 C1–2	Clinical signs: dyspnea, paralysis, convulsions, jump, respiratory failure, and death. In necropsy a pronounced isquemic zone only in liver border was detected. Histopathological changes: cerebellar injury (Purkinje cell degeneration).	[110]
Crustaceans	*Acartia clausi*	GCCV-14	f/2 + Se 10^−8^M, 33– 34‰, 20 ºC, 12:12 L:O, 150 μEm^2^ s^−1^, 2 L flasks	60	dcSTX, dcGTX-2, 3,C1–2	No adverse effects.	[101]
	*Litopenaeu s vannamei*	GCCV-6	f/2, 33‰, 26 ± 1 ºC, 12:12 L:O, 150 μEm^2^ s^−1^, Fernbach flasks	nd	STX, dcSTX, neoSTX, GTX-1,2,3,4	Paralysis of antennae and pereiopods, disequilibrium, atypical swimming. Slow and irregular movements of gills, pleopods, and maxillipeds. Heart and brain severely damaged; juvenile shrimp more susceptible than adult animals. In chronic exposure: gastric glands and muscle retained paralytic toxins for a longer period, histological damages were observed in the heart, gastric gland, and brain tissue.	[111,112]
Mollusks	*Artemia salina* (adults and metanauplii)		GSe, 33‰, 23 ± 1 ºC, 12:12 L:O, 150 μEm^2^ s^−1^, Fernbach flasks	nd	STX, dcSTX GTX-2,3, dcGTX-2,3 C1–2	20% and 10% mortality in adults and metanauplii, respectively. Clinical signs: Adults: erratic swimming (circles), spasms, convulsions, and death. Metanauplii: erratic swimming and death.	this study
	*Nodipecten subnodosus*		GSe medium, 32‰, 21 ºC, 16:8 L:O, 70 W, 20 L flasks	2–5	GTX	At high food concentrations, juvenile showed production of pseudofeces, partial shell valve closure, and reduction in feeding. An increase of antioxidant and hydrolytic enzymes mainly in gills and the digestive gland. Melanization in gills, mantle, and labial palps.	[115,116]
	*Megapitaria squalida*	GCCV-7	f/2 + Se 10^−8^M, 33–34‰_,_ 22 ºC, 12:12 L:O, 150 μEm^2^ s^−1^, 10 L flasks	26–28	STX, dcSTX, neoSTX, dcGTX-3, 4, C1–2	No adverse effects.	[108]

BACO, Bahía Concepción; BAPAZ, Bahía de La Paz; BAMAZ, Bahía de Mazatlán. nd, not detected. References [17,101].

**Table 6:** Growth rate and maximum cell density of *Gymnodinium catenatum* strains of the Gulf of California in different growth conditions.

Strain Code	Source	Growth Rate (div day^−1^)	Maximum Cell Density (Cells mL^−1^)	Media	Temperature (ºc)	Salinity (psu)	Light Intensity (μmol m^2^ s^−1^)	Light/Dark Cycle	Ref.
GCCQ-1		0.74 ± 0.07	1619 ± 252						
GCCV-2	BACO	0.70 ± 0.07	1090 ± 270						
GCCV-4		0.82 ± 0.09	3393 ± 836						
GCPV-1	BAPAZ	0.74 ± 0.06	1631 ± 152	GSe	20 ± 1	35	150	12,12	[104]
GCPV-2		0.77 ± 0.05	1421 ± 290						
GCMV-1	BAMAZ	0.81 ± 0.02	2063 ± 226						
GCMV-2		0.82 ± 0.03	1865 ± 516						
		0.14–0.21	nd	f/2 + Se 10^−8^ M	15–29	30	230	10,14	
		0.24	nd			26–30	150	10,14	
GCCV-10	BACO	0.28–0.31	nd		20	28–38	150	12,12	[103]
		0.15–0.19	1559–1970	f/2 + Se 10^−6^, 10^−7^, 10^−8^ M		35	150	12,12	
